# Nutritional status and dietary diversity of pregnant and nonpregnant reproductive‐age Rohingya women

**DOI:** 10.1002/fsn3.3508

**Published:** 2023-06-15

**Authors:** Shah Mohammad Fahim, Subhasish Das, Md. Golam Rasul, Mahabub Uz Zaman, Md. Ashraful Alam, Sazia Afrin, Kazi Nazmus Saqeeb, Md. Mahmudul Hasan, A. F. M. Mahbubul Alam, Morseda Chowdhury, Tahmeed Ahmed

**Affiliations:** ^1^ Nutrition and Clinical Services Division International Centre for Diarrhoeal Disease Research, Bangladesh (icddr,b) Dhaka Bangladesh; ^2^ Division of Nutritional Sciences Cornell University Ithaca New York USA; ^3^ Health, Nutrition and Population Programme BRAC Dhaka Bangladesh; ^4^ Office of the Executive Director, International Centre for Diarrhoeal Disease Research, Bangladesh (icddr,b) Dhaka Bangladesh; ^5^ Department of Global Health University of Washington Seattle Washington USA; ^6^ Department of Public Health Nutrition, James P Grant School of Public Health BRAC University Dhaka Bangladesh

**Keywords:** Bangladesh, dietary diversity, double burden of malnutrition, overnutrition, pregnancy, refugee health, Rohingya, undernutrition, women of reproductive age

## Abstract

There are no data on the nutritional status and dietary diversity of the pregnant and nonpregnant reproductive‐age Rohingya women who have recently shifted to the Bhasan Char Relocation Camp located on an island in the Bay of Bengal. A cross‐sectional survey was conducted in November–December, 2021 to assess the nutritional status and evaluate the dietary diversity of two vulnerable groups of the forcibly displaced Rohingya population: nonpregnant reproductive‐age women and pregnant mothers. Multivariable logistic regression was applied to identify the factors associated with nutritional impairments. Overall, 7.6% of the nonpregnant reproductive‐age women were underweight (Body Mass Index [BMI] < 18.5 kg/m^2^), and nearly one‐third of them had a BMI ≥ 25 kg/m^2^. However, 26.7% of the pregnant mothers were undernourished (BMI < 20.0 kg/m^2^) and almost one‐fourth of them were either overweight or obese (BMI ≥ 25.0 kg/m^2^). The prevalence of thinness (Mid Upper Arm Circumference [MUAC] < 23 cm) was 34.5% among pregnant mothers, and 10.1% of them were severely thin (MUAC < 21 cm). The mean (±SD) of the Women's Dietary Diversity Score (WDDS) was 3.3 (±1.1) for nonpregnant reproductive‐age women and 3.7 (±1.3) for pregnant mothers enrolled in this study. Overall, 63.8% of the nonpregnant women of childbearing age and 46% of the pregnant mothers had a low WDDS (WDDS < 4). The WDDS was found to be protective against thinness among nonpregnant reproductive‐age women (AOR = 0.61; 95% CI = 0.37, 0.93; *p*‐value = .03) and low BMI in pregnant mothers (AOR = 0.71; 95% CI = 0.55, 0.91; *p*‐value = .01). The results of this survey will assist in early recognition of the nutritional demands, and act as a guide to planning nutrition‐based programs among Rohingya reproductive‐age women relocated to the Bhasan Char Island.

## Graphical abstract

A cross‐sectional survey was conducted in November–December, 2021 to assess the nutritional status and the dietary diversity of the forcibly displaced Rohingya women of reproductive age. The results of this survey will assist in early recognition of the nutritional demands, and act as a guide to planning nutrition‐based programs among Rohingya reproductive‐age women relocated to the Bhasan Char.

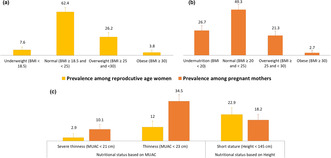

## 1 INTRODUCTION

The forceful displacement of the Rohingya population from the Rakhine state of Myanmar is considered a major humanitarian crisis globally (Mukul et al., 2019). The violence escalated in August 2017 resulting in an influx of approximately 700,000 Rohingya population to the neighboring country Bangladesh (Leidman et al., 2020). This huge number of the population joined around 200,000 Rohingya people who fled earlier over a period of three decades (Leidman et al., 2018). Currently, nearly a million Rohingya people live in different camps and makeshift settlements in Cox's Bazar district of Bangladesh (Zaman et al., 2019). The Government of Bangladesh has been relocating the Rohingya population to Bhasan Char Island in the Bay of Bengal since October 2017. Over the past 4 years, 17,698 Rohingya individuals have been shifted to the Bhasan Char Relocation Camps. Among them, 1016 are pregnant mothers. It is known that women, adolescent girls, and children suffer the most in any kind of conflict, and the Rohingya refugee community is no exception (Yousuf et al., 2020; Zaman et al., 2019). According to recent information, almost half of the reproductive‐age women living in Bhasan Char are suffering from anemia (Joarder et al., 2020). However, there is a lack of data for other nutritional indices among pregnant mothers and nonpregnant women of reproductive age. While no information is available regarding the food security and dietary diversity of Rohingya women who shifted to Bhasan Char, surveys conducted in the refugee camps of Cox's Bazar reported persistent food insecurity and nutritional deficiencies among the same population (Khan et al., 2020; Mukul et al., 2019). As Rohingya women of reproductive age are being transferred to Bhasan Char from the refugee camps of Cox's Bazar, it is imperative to assess their nutritional status as well as dietary practices to facilitate the development of appropriate public health programs for this vulnerable group of population living on a geographically isolated island. Detailed information regarding their current nutritional status and dietary habit is also essential to designing and adopting effective nutrition‐related intervention programs for both pregnant and nonpregnant Rohingya women of reproductive age living on Bhasan Char Island. Therefore, we have designed this study to assess the nutritional status and dietary diversity among Rohingya women of reproductive age and pregnant mothers living in the Bhasan Char of Bangladesh. The findings of this study will generate evidence on the nutritional status of pregnant mothers, and nonpregnant women of reproductive age living in the Bhasan Char Relocation Camp and help the policymakers to design appropriate public health programs for this population.

## 2 METHODS

### 2.1 Ethics statement

The study protocol was approved by the Office of the Refugee Relief and Repatriation Commissioner (RRRC), Bangladesh, and the Institutional Review Board of the International Centre for Diarrhoeal Disease Research, Bangladesh (icddr,b), Dhaka, Bangladesh. The study also conformed to the Declaration of Helsinki. Participation in the study was entirely voluntary, and participants were informed about risks and benefits of the study prior to enrolment. Informed written consent was obtained from the study participants before data collection. The privacy and anonymity of the participants were also strictly maintained in the study.

### 2.2 Study design and settings

It was a cross‐sectional survey conducted in November–December, 2021 between two vulnerable groups of the Rohingya population: nonpregnant women of reproductive age and pregnant mothers. The study location was the Bhasan Char Relocation Camp for the Rohingya Refugee Population. Bhasan Char is an island located in the Bay of Bengal, and administratively a part of Hatiya Upazila of Noakhali District in Bangladesh. The island is about 6 km from Sandwip Island and approximately 37 miles from the mainland of Bangladesh. During the data collection period, 17,698 forcibly displaced Rohingyas were staying in 18 shelters of Bhasan Char Relocation Camp. Each shelter had 192 households. Around 1016 pregnant women and 3000 nonpregnant women of reproductive age were living in these accommodations.

### 2.3 Sample size

The sample size was calculated using the formula for cross‐sectional study design [*n* = *Z* ^2^ × *p*(1 − *p*)/*d* ^2^]. We have considered the underweight (BMI < 18.5) prevalence for nonpregnant women of reproductive age. The prevalence of thinness (MUAC < 23) was used for the sample size calculation of pregnant mothers. As per recent data, 16% of the Rohingya women of reproductive age were underweight (BMI < 18.5) (Chowdhury et al., 2018), and the prevalence of thinness among pregnant mothers was 25% (Corna et al., 2019). Using these above‐mentioned proportions, the minimum required sample sizes for women of reproductive age and pregnant mothers were 207 and 289, respectively. A sampling frame was created from the available household list received from the office of the camp in charge. The study participants were selected randomly using computer‐generated random numbers. The households were selected in such a way that every participant represents a unique household.

### 2.4 Data collection

A sampling frame was created from the available household list provided by the RRRC office at Bhashan Char relocation camp. Computer‐generated random numbers were generated to select the participant's household randomly. Only one participant was enrolled from each household. Sociodemographic information and anthropometry data (height, weight, and Mid‐Upper Arm Circumference [MUAC]) were collected from the study participants at enrolment. Trained research staff measured anthropometry using validated anthropometric tools (Seca 217 Stadiometer for height, TANITA HD‐662 Digital Scale for weight, and Adult MUAC Tape for MUAC). Body Mass Index (BMI) was calculated from the height and weight of the recruited individuals. In addition, dietary data were collected using the Food Frequency Questionnaire (FFQ). The FFQ consists of 16 questions and was developed based on the guidelines from the Food and Agriculture Organization (FAO, 2018; Kennedy et al., 2013). The dietary data were combined to calculate the Women's Dietary Diversity Score (WDDS) (Kennedy et al., 2011).

### 2.5 Operational definitions

Nutritional status was assessed based on BMI, MUAC, and height of the participants. For nonpregnant reproductive age women, BMI was categorized as follows: Underweight = BMI <18.5 kg/m^2^, Normal = BMI ≥18.5 and <25.0 kg/m^2^, Overweight = BMI ≥25.0 kg/m^2^ and <30.0 kg/m^2^, and Obese = BMI ≥30.0 kg/m^2^ (National Institute of Population Research and Training [NIPORT] & ICF, 2020; WHO, 1995). The criteria we followed for pregnant mothers are as follows: Underweight = BMI <20.0 kg/m^2^, Normal = BMI ≥20.0 and <25.0 kg/m^2^, Overweight = BMI ≥25.0 and <30.0 kg/m^2^, and Obese = BMI ≥30.0 kg/m^2^ (Rayis et al., 2010). Severe thinness was defined if the MUAC was less than 21 cm, while thinness was defined as a MUAC of less than 23 cm (Assefa et al., 2012; Gebre et al., 2018). The cut‐off used for short stature was 145 cm for both groups (National Institute of Population Research and Training [NIPORT] & ICF, 2020; Toh‐Adam et al., 2012). In addition, a WDDS of less than 4 was considered a low dietary diversity among the study participants (Zerfu et al., 2016).

### 2.6 Statistical analysis

All the statistical analyses were performed using R version 4.0.4 (https://www.r‐project.org; Foundation for Statistical Computing) software. Normally distributed numerical variables were summarized using mean and standard deviation. Median and interquartile ranges were used for the variables following skewed distributions. Binary and categorical variables were presented as frequencies and percentages. The Appropriate statistical tests (Student's *t*‐tests, Pearson's Chi‐square tests, and Mann–Whitney test) were used to detect any group‐wise differences. Multivariable logistic regression analyses were done to identify the factors associated with the nutritional status of the study participants. At first, univariate regression analyses were done, and variables with a *p*‐value <0.20 were included in the multivariable models. In addition, the models were adjusted for the age of the participants. A probability of <.05 was considered statistically significant.

## 3 RESULTS

Overall, 210 women of reproductive age and 296 pregnant mothers were enrolled in this study. The mean age (±SD) of the participants was 29.62 (±7.36) years and 24.22 (±5.78) years, respectively. Most of the study participants never attended school. However, all the enrolled participants had provision of improved water and sanitation. The basic and demographic characteristics of the study participants are reported in Table 1.

**TABLE 1:** Descriptive characteristics of the study participants.

Variables	Nonpregnant women of reproductive age (*n* = 210)	Pregnant mothers (*n* = 296)
Age in years, mean ± SD	29.62 ± 7.36	24.22 ± 5.78
School years, median (Q1, Q3)	0 (0, 2)	0 (0, 0)
Number of family members, median (Q1, Q3)	5 (3, 6)	4 (3, 5)
Monthly family income in BDT, median (Q1, Q3)	1800 (1400, 3000)	2000 (1400, 5000)
Average monthly expenditure of the entire household for food in BDT, median (Q1, Q3)	2000 (1400, 3000)	3000 (2000, 5000)
Improved source of drinking water, *n* (%)	210 (100)	296 (100)
Sanitation (flush to septic tank), *n* (%)	210 (100)	296 (100)
Treat water to make it safer to drink, *n* (%)	161 (76.67)	243 (82.09)
Crowding, *n* (%)		
High	20 (9.5)	32 (10.8)
Low	190 (90.5)	264 (89.2)
Weight in kg, mean ± SD	52.09 ± 8.60	51.12 ± 9.40
Height in cm, mean ± SD	149.13 ± 5.11	150.40 ± 5.95
Mid‐upper arm circumference (MUAC) in cm, Mean ± SD	26.42 ± 3.00	24.58 ± 3.12
Body mass index (BMI), mean ± SD	23.38 ± 3.46	22.57 ± 3.70
Wash hands with soap before preparing food, *n* (%)		
Never	13 (6.19)	21 (7.09)
Sometimes	154 (73.33)	128 (43.24)
Always	43 (20.48)	147 (49.66)
Wash hands with soap before eating, *n* (%)		
Never	7 (3.33)	5 (1.69)
Sometimes	155 (73.81)	105 (35.47)
Always	48 (22.86)	186 (62.84)
Wash hands with soap after using the toilet, *n* (%)		
Never	12 (5.71)	3 (1.01)
Sometimes	147 (70.00)	60 (20.27)
Always	51 (24.29)	233 (78.72)
Age at marriage, mean ± SD	16.99 ± 2.12	17.04 ± 2.62
Age at first pregnancy, mean ± SD	18.34 ± 2.31	18.20 ± 2.51
Pregnancies had in her lifetime, median (Q1, Q3)	4 (2, 5)	2 (1, 4)
Live birth had in her lifetime, median (Q1, Q3)	3 (2, 5)	2 (1, 3)
Betel leaf and nuts, *n* (%)	127 (60.48)	118 (39.86)
Tobacco, *n* (%)	68 (32.38)	60 (20.27)
Smoking, *n* (%)	4 (1.90)	11 (3.72)

### 3.1 Nutritional status of the study participants

**FIGURE 1:**
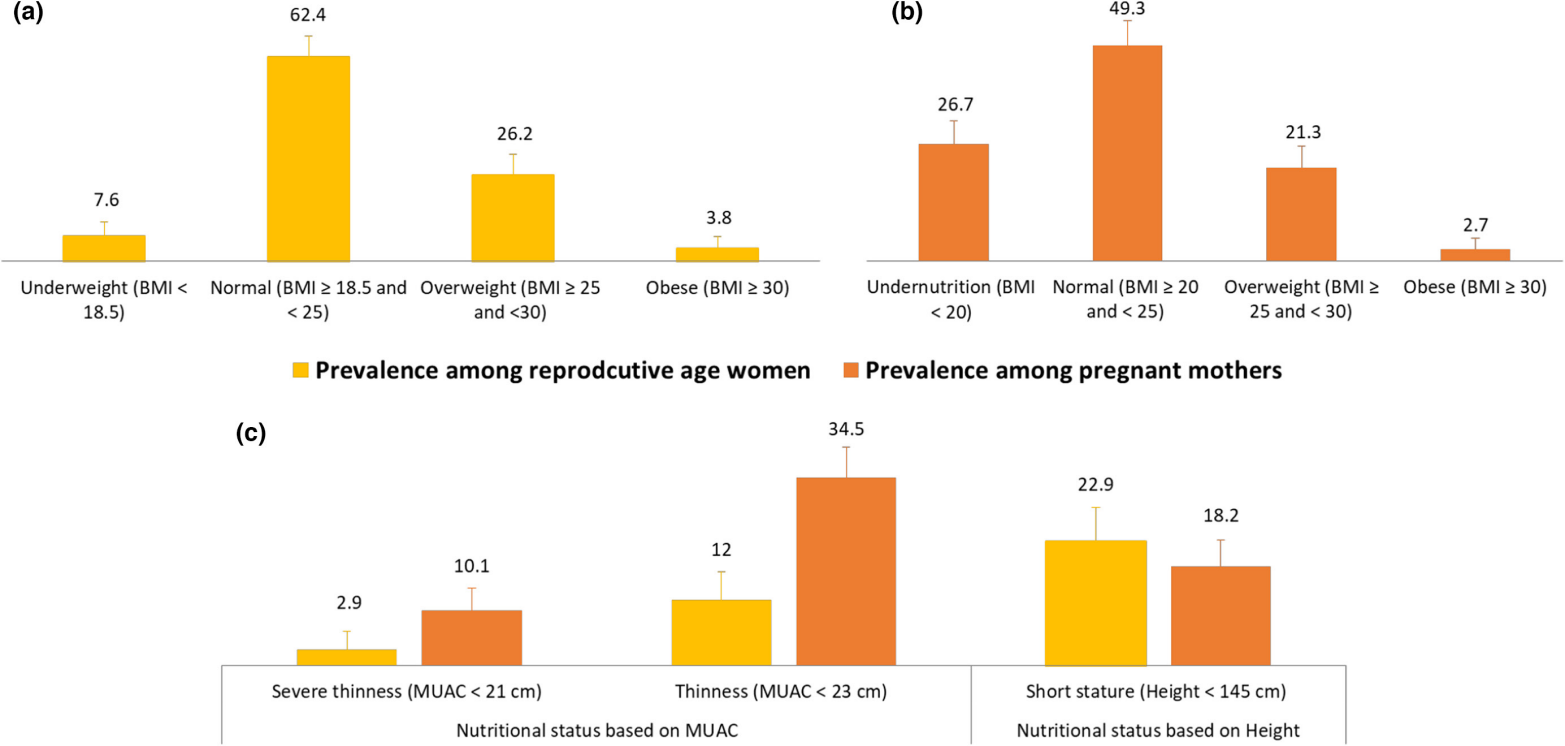
Nutritional status of the study participants.

The nutritional status of the study participants is displayed in Figure 1. The prevalence of underweight (BMI < 18.5 kg/m^2^) was 7.6% among nonpregnant women of reproductive age. However, the prevalence of overweight and obesity among the same group was 26.2% and 3.8%, respectively. Overall, 2.9% of the nonpregnant women were severely thin, and 12.0% had a MUAC of less than 23 cm. The prevalence of short stature in nonpregnant women was 22.9%. Overall, 26.7% of the pregnant mothers had a BMI of less than 20 kg/m^2^, 21.3% were overweight, and 2.7% were obese. The prevalence of thinness was 34.5% among pregnant mothers, and 10.1% of the pregnant women were severely thin. We observed an 18.2% prevalence of short stature among the pregnant mothers enrolled in this study.

### 3.2 Dietary diversity among the study participants

**FIGURE 2:**
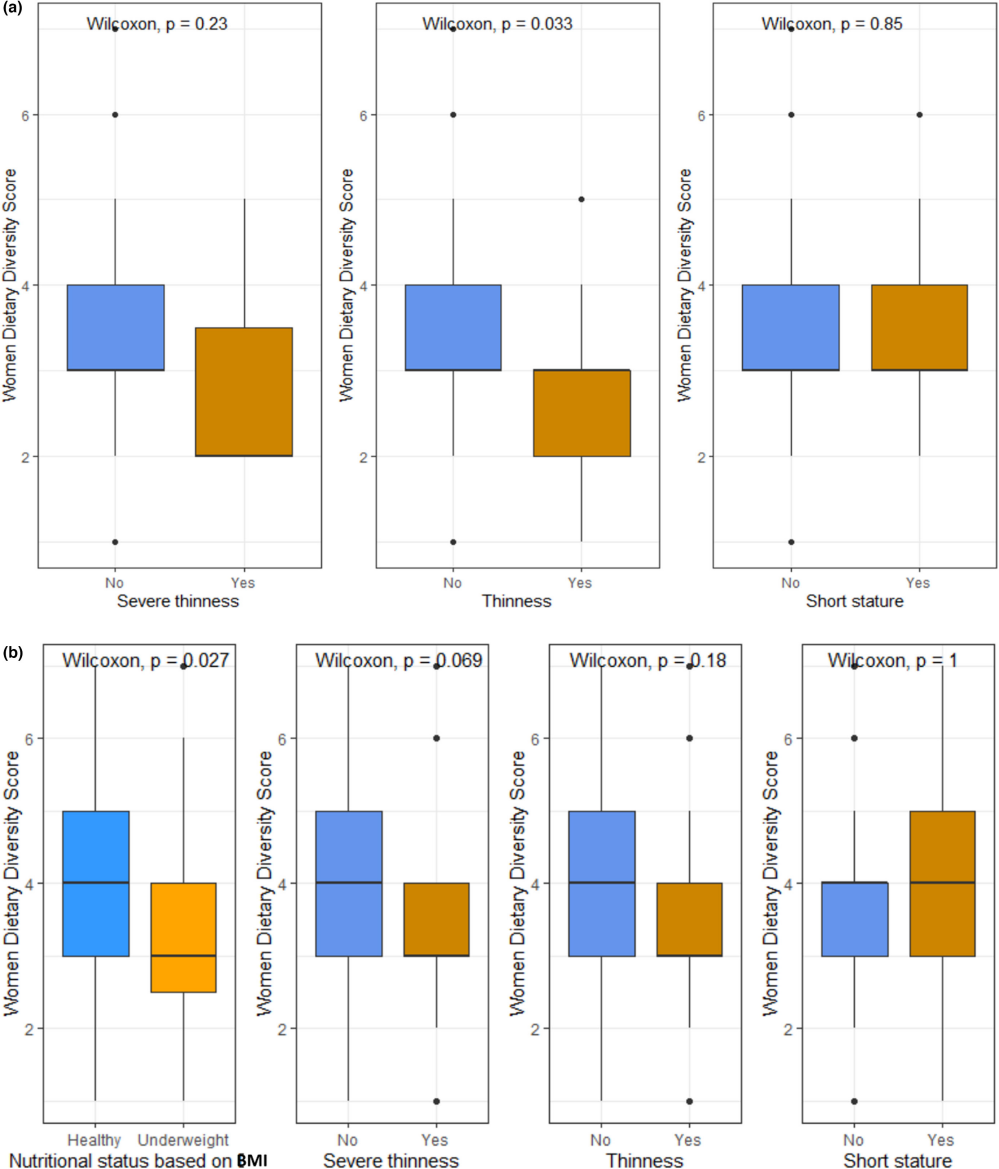
The differences in WDDS based on the nutritional status of the study participants.

The mean (±SD) WDDS was 3.3 (±1.1) for nonpregnant reproductive‐age women and 3.7 (±1.3) for pregnant mothers enrolled in this study. Overall, 63.8% of the nonpregnant women of reproductive age and 46% of the pregnant mothers had a low WDDS (WDDS < 4). Figure 2 demonstrates the differences in WDDS based on the nutritional status of the study participants. A significant difference in WDDS has been observed between thin and nonthin women of reproductive age (*p*‐value = .033). We also observed that WDDS was high among healthy pregnant mothers compared to underweight women with pregnancy (*p*‐value = .027).

The proportion of different food groups consumed by the study participants is shown in Figure S1. Consumption of starchy staples, oils, fats, spices, and condiments was almost 90% in both study groups. Nearly half of the women (46.2% for nonpregnant reproductive age women and 55.7% for pregnant mothers) consumed meat and fish. Consumption of milk and eggs was very low among the study participants. Only 5.7% of the reproductive‐age women and 11.5% of pregnant mothers consumed eggs, and the proportions for milk and milk products are 1.4% and 2.7%, respectively. More than 80% of the participants consumed vitamin A‐rich fruits and vegetables. However, the consumption of dark green leafy vegetables was very minimal in both groups (15.2% and 26.4%, respectively).

### 3.3 Factors associated with thinness among pregnant and nonpregnant reproductive‐age women

Table 2 shows the factors associated with thinness in nonpregnant women of reproductive age. WDDS was inversely associated with thinness in nonpregnant women of reproductive age after adjusting for age, education, and presence of a source of family income, and the association was statistically significant (AOR = 0.61; 95% CI = 0.37, 0.93; *p*‐value = .03). However, neither WDDS nor any other factors were found to be associated with thinness among pregnant mothers in multivariable regression analysis.

**TABLE 2:** Factors associated with thinness among nonpregnant women of reproductive age living in Bhasan Char Relocation Camp.

Factors	OR (95% CI)	*p*‐Value	AOR (95% CI)	*p*‐Value
Age in years	0.97 (0.91, 1.03)	.30	0.94 (0.88, 1.00)	.08
Education (received)	0.40 (0.13, 1.04)	.08	0.35 (0.11, 0.96)	.06
Source of income in the family (present)	0.28 (0.04, 0.99)	.09	0.28 (0.04, 1.06)	.10
Women's Dietary Diversity Score	0.58 (0.36, 0.91)	.02	0.61 (0.37, 0.93)	.03

*Note*: Multivariable logistic regression model adjusted for the variables with *p*‐values <.20 in the univariate logistic regression analysis.

Abbreviations: AOR, adjusted odds ratio; CI, confidence interval; OR, odds ratio.

### 3.4 Factors associated with underweight, overweight, and obesity in pregnant and nonpregnant Rohingya women

Table 3 demonstrates the results of multivariable logistic regression analysis for factors associated with underweight, overweight, and obesity in pregnant mothers living on Bhasan Char island. We observed that high WDDS was protective against low BMI (AOR = 0.71; 95% CI = 0.55, 0.91; *p*‐value = .01) in pregnant mothers after adjusting for age, number of pregnancies, number of live births, and education of the participants. The WDDS was also inversely associated with being overweight in pregnant women and this finding was marginally significant (AOR = 0.78; 95% CI = 0.60, 1.01; *p*‐value = .06). We observed a statistically significant positive association of age with overweight (AOR = 1.17; 95% CI = 1.08, 1.28; *p*‐value <.001) and obesity (AOR = 1.20; 95% CI = 1.03, 1.39; *p*‐value = .02) among the pregnant mothers. However, no significant finding was found for nonpregnant Rohingya women in multivariable logistic regression analysis.

**TABLE 3:** Factors associated with underweight, overweight, and obesity in pregnant mothers living in Bhasan Char Relocation Camp.

Factors	Underweight (BMI <20.0 kg/m^2^)			Overweight (BMI ≥25.0 and <30.0 kg/m^2^)			Obesity (BMI ≥30.0 kg/m^2^)		
	AOR	95% CI	*p*‐Value	AOR	95% CI	*p*‐Value	AOR	95% CI	*p*‐Value
Age in years	1.05	0.96, 1.15	.32	1.17	1.08, 1.28	<.001	1.20	1.03, 1.39	.02
Number of pregnancies	1.23	0.53, 2.83	.63	0.83	0.39, 1.74	.62	1.04	0.24, 4.60	.96
Number of live births	0.59	0.24, 1.44	.25	1.11	0.51, 2.42	.80	0.77	0.15, 3.86	.75
Education (received)	2.04	0.94, 4.44	.07	2.84	1.24, 6.51	.01	0.98	0.10, 9.25	.99
Women's Dietary Diversity Score	0.71	0.55, 0.91	.01	0.78	0.60, 1.01	.06	1.43	0.81, 2.52	.22

*Note*: Multivariable logistic regression model adjusted for the variables with *p*‐values <.20 in the univariate logistic regression analysis.

Abbreviations: AOR, adjusted odds ratio; BMI, body mass index; CI, confidence interval.

## 4 DISCUSSION

Our results demonstrate that both under‐ and overnutrition are extant among the Rohingya women of reproductive age who moved to the Bhasan Char Island. Although the prevalence of undernutrition in nonpregnant women was estimated less than 10%, nearly one‐third of them were diagnosed as overweight or obese. More than one‐fourth of the pregnant mothers enrolled in this study were undernourished. On the other hand, a significant proportion of the women with pregnancy were overweight or obese. The prevalence of thinness among pregnant women was almost 35%, and more than 10% of them were severely thin with a MUAC of less than 21 cm. Almost one‐fifth of the pregnant mothers were short for their height, and 23% of Rohingya women of child‐bearing age had short stature. However, the prevalence of underweight was lower compared to the national prevalence in Bangladesh. As per the Bangladesh Demographic and Health Survey 2017–18, 11.9% of the women of reproductive age are underweight (National Institute of Population Research and Training [NIPORT] & ICF, 2020). This finding ascertains the improvement in the nutritional status of the Rohingya women. Effective public health interventions in addition to increased coverage of food rations, distributions of fortified foods, and supplementation with micronutrient powders are the key drivers of such improvement in the nutritional status of this vulnerable population. However, there need additional efforts to ameliorate the nutritional status of pregnant mothers. We observed that both the prevalence of thinness and low BMI were substantial among the Rohingya women with pregnancy. Optimal nutrition during pregnancy is important because nutritional impairments during this critical period may lead to a vicious cycle of obstetric complications including poor neonatal outcomes, growth failure, childhood malnutrition, and subsequent adversity during the adult lifetime (Fahim et al., 2020). Low BMI and a MUAC <23 cm among pregnant mothers increase the risk of intrauterine growth retardation (IUGR). Hence, special attention is required in the management of nutritional impairments of pregnant mothers shifted to Bhasan Char.

The prevalence of short stature was much higher among Rohingya women compared to the national prevalence in Bangladesh women (National Institute of Population Research and Training [NIPORT] & ICF, 2020). The short stature among the Rohingya women can be constitutional or may be a reflection of chronic food insecurity during childhood (Schmeer & Piperata, 2017; Weigel et al., 2016). Prior works revealed that insufficient weight gain during early childhood correlates with shorter height in later life (Koyama et al., 2022). Short stature may lead to complications during pregnancy (Sohlberg et al., 2012). Additionally, maternal height is an important determinant of childhood growth (Mal‐Ed Network Investigators, 2017). Therefore, it is imperative to concentrate on the nutritional requirements of female children during the early years of life to improve their linear growth.

We observed a higher proportion of overweight and obese women both in pregnant and nonpregnant groups. This result is in accordance with the findings from different refugee camps around the world (Belau et al., 2021; Damiri et al., 2018; Eryurt & Menet, 2020; Grijalva‐Eternod et al., 2012). Perhaps, limited space and scope for physical activity are primarily responsible for this phenomenon (Andersen et al., 2021; Eryurt & Menet, 2020). In addition, food insecurity plays a crucial role in the development of overweight and obesity among the refugee population (Abou‐Rizk et al., 2021). Low dietary diversity and limited access to nutritious food lead to the consumption of energy‐dense nonnutritious meals which ultimately result in excessive weight gain (Jomaa et al., 2017). Evidence suggests that lack of diversity in food consumption and inadequate intake of specific food groups are the key drivers for coexistence of both under‐ and overnutrition in refugee settlements (Khuri et al., 2022).

Consistent with this notion, our results revealed that dietary diversity was poor among the Rohingya women of both groups. Dietary diversity is a proxy indicator of nutrient adequacy at individual levels (Oldewage‐Theron & Kruger, 2011). Earlier works showed that adults with low dietary diversity scores are more likely to be undernourished (Fahim et al., 2020; Nithya & Bhavani, 2018). It was also reported that poor dietary diversity during pregnancy results in micronutrient deficiencies, low birth weight, and unfavorable birth outcomes (Bitew et al., 2021; Rammohan et al., 2019). We found that consumption of dairy products and eggs was very minimal among the Rohingya women. The intake of protein‐rich food items was also suboptimal among the enrolled participants. Moreover, the consumption of dark green leafy vegetables, sources of iron and folate, was not satisfactory to meet the requirements of women of childbearing age. Maternal iron and folate deficiencies are important determinants of adverse pregnancy outcomes (Park et al., 2012). Therefore, attention needs to be given to the food package being supplied to the Rohingya population in Bhasan Char. The existing food package consists of nondiversified starch‐based food items (Table S1). The inclusion of eggs, dairy products, and local produce in the package can be a potential option to improve the dietary diversity of this population.

### 4.1 Strengths and limitations of the study

This study has multiple strengths. First, this is the first study to assess the nutritional status and dietary diversity of both pregnant and nonpregnant Rohingya refugee women shifted to a remote island in the Bay of Bengal. Second, we have used validated tools to measure the nutritional status and dietary diversity of this vulnerable group of population. Third, the brief period of data collection limited the chances of variability in dietary diversity due to seasonality, demographic characteristics, and political instability. However, the study has several limitations as well. First, we could not collect data on biochemical markers and micronutrient status of the Rohingya women which is one of the major limitations of the study. Second, the assessment of nutritional status using BMI has inherent methodological challenges. However, BMI is widely accepted and appropriate for application in limited resource settings including make‐shift refugee camps. Third, dietary data were collected only once, and based on 24‐h dietary recall method. It is, therefore, possible that we might have failed to document the regular dietary practice of some of the participants. Fourth, the lack of clinical and morbidity data are another important limitation of this study. Finally, the cross‐sectional nature of the study limited our ability to investigate the causal factors of nutritional impairments in pregnant and nonpregnant Rohingya women of reproductive age.

## 5 CONCLUSIONS

Our results suggest that both under‐ and overnutrition are prevalent among forcibly displaced Rohingya women irrespective of their pregnancy status. We also observed poor dietary diversity scores among the same population. The study findings warrant development of tailored nutrition‐related intervention programs to avert the consequences of intraindividual and household‐level double burden of malnutrition in this settlement. In addition to that, culturally appropriate public health programs should be designed to ensure optimum nutritional status and dietary diversity among the Rohingya refugee women of childbearing age. We believe that the results of this survey will assist the policymakers in early recognition of the nutritional demands, and act as a guide to planning nutrition‐based programs for Rohingya refugee women. However, future longitudinal studies are required to identify the key drivers influencing the incidence of both under‐ and overnutrition among this vulnerable group of population transferred to Bhasan Char Island.

## AUTHOR CONTRIBUTIONS

**Shah Mohammad Fahim:** Conceptualization (lead); formal analysis (lead); investigation (lead); methodology (lead); project administration (lead); resources (lead); software (lead); supervision (lead); writing – original draft (lead); writing – review and editing (equal). **Subhasish Das:** Conceptualization (equal); methodology (equal); project administration (equal); resources (equal); supervision (equal); writing – review and editing (equal). **Md. Golam Rasul:** Data curation (equal); formal analysis (equal); project administration (equal); writing – review and editing (equal). **Mahabub Uz Zaman:** Data curation (equal); formal analysis (equal); project administration (equal); writing – review and editing (equal). **Md. Ashraful Alam:** Data curation (equal); formal analysis (equal); software (equal); writing – review and editing (equal). **Sazia Afrin:** Writing – original draft (equal); writing – review and editing (equal). **Kazi Nazmus Saqeeb:** Project administration (equal); writing – review and editing (equal). **Md. Mahmudul Hasan:** Project administration (equal); writing – review and editing (equal). **A. F. M. Mahbubul Alam:** Project administration (equal); writing – review and editing (equal). **Morseda Chowdhury:** Conceptualization (equal); methodology (equal); project administration (equal); resources (equal); supervision (equal); writing – review and editing (equal). **Tahmeed Ahmed:** Conceptualization (equal); investigation (equal); methodology (equal); project administration (equal); resources (equal); supervision (equal); writing – review and editing (equal).

## FUNDING INFORMATION

No specific fund has been obtained for this study. The study activities were done using the existing setup and facilities of the International Centre for Diarrhoeal Disease Research, Bangladesh (icddr,b), and BRAC, Bangladesh.

## CONFLICT OF INTEREST STATEMENT

The authors have declared that no competing interests exist.

## Supporting information


Figure S1

Table S1

## Data Availability

Data related to this manuscript are available upon request, and researchers who meet the criteria for access to confidential data may contact Ms. Armana Ahmed (armana@icddrb.org) at the Research Administration of icddr,b (http://www.icddrb.org/).
