# Supplementary figures and images for: Nutritional status and dietary diversity of pregnant and nonpregnant reproductive‐age Rohingya women
Source: Food Sci Nutr. 2023 Jun 15;11(9):5523–31. doi: 10.1002/fsn3.3508 (PMC10494628; doi:10.1002/fsn3.3508)

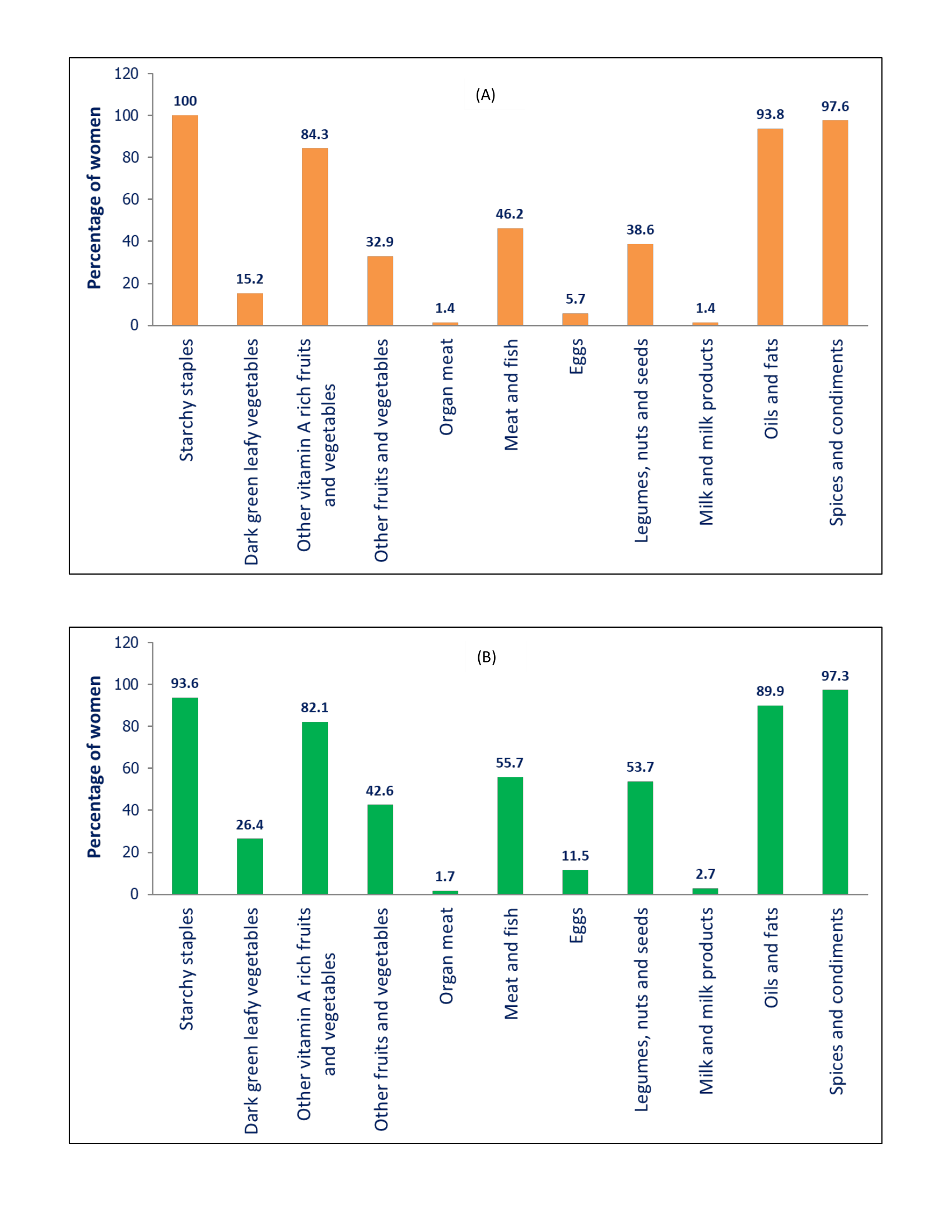

Supplement: Supplementary file 1 — Figure S1 [file FSN3-11-5523-s002.tiff]
