# Supplementary material for: Nutritional status and dietary diversity of pregnant and nonpregnant reproductive‐age Rohingya women
Source: Food Sci Nutr. 2023 Jun 15;11(9):5523–31. doi: 10.1002/fsn3.3508 (PMC10494628; doi:10.1002/fsn3.3508)
Supplement: Supplementary file 2 — Table S1 [file FSN3-11-5523-s001.docx]

**Supplementary Table 1.** Food items per month for the Rohingya population shifted to Bhasan Char Relocation Camp

| **Food items** | **Amount based on family Size** | | | | |
| --- | --- | --- | --- | --- | --- |
|  | **2 people** | **3 people** | **4 people** | **5 people** | **6 people** |
| Rice | 26 kg | 39 kg | 52 kg | 65 kg | 78 kg |
| Lentil | 2 kg | 2 kg | 3 kg | 4 kg | 5 kg |
| Oil | 2 liters | 3 liters | 4 liters | 5 liters | 5 liters |
| Salt | 500 g | 1 kg | 1 kg | 1 kg | 1.5 kg |
| Sugar | 1 kg | 2 kg | 2 kg | 3 kg | 3 kg |
| Onion | 2 kg | 2.5 kg | 3 kg | 4 kg | 5 kg |
| Garlic | 400 g | 500 g | 600 g | 800 g | 1 kg |
| Ginger | 200 g | 300 g | 400 g | 500 g | 600 g |
| Turmeric powder | 200 g | 200 g | 200 g | 250 g | 300 g |
| Chili powder | 250 g | 250 g | 350 g | 400 g | 500 g |
| Coriander powder | 100 g | 150 g | 200 g | 250 g | 300 g |
| Cumin powder | 100 g | 100 g | 150 g | 200 g | 200 g |
